# Treatment of resistant metastatic melanoma using sequential epigenetic therapy (decitabine and panobinostat) combined with chemotherapy (temozolomide)

**DOI:** 10.1007/s00280-014-2501-1

**Published:** 2014-07-26

**Authors:** Chang Xia, Roberto Leon-Ferre, Douglas Laux, Jeremy Deutsch, Brian J. Smith, Melanie Frees, Mohammed Milhem

**Affiliations:** 1 Division of Hematology, Oncology and Bone and Marrow Transplantation, Department of Internal Medicine, University of Iowa Hospitals and Clinics, 200 Hawkins Drive, Iowa City, IA 52242 USA; 2 Department of Internal Medicine, University of Iowa Hospitals and Clinics, 200 Hawkins Drive, Iowa City, IA 52242 USA; 3 Oncology-Hematology, St Mary’s Health Center, Richmond Heights, MO USA; 4 Oncology-Hematology, Capital Region Medical Center, 1241 W Stadium Blv, Jefferson City, MO 65109 USA; 5 Oncology-Hematology, Cancer Center of Kansas, 2322 N Chelmsford, Wichita, KS 67228 USA; 6 Department of Biostatistics, College of Public Health, University of Iowa, 105 River St, Iowa City, IA 52242 USA

**Keywords:** Melanoma, Epigenetics, Epigenetic priming, Resistance, Hypomethylation, Histone deacetylation

## Abstract

**Purpose:**

To explore the safety and tolerability of combining two epigenetic drugs: decitabine (a DNA methyltransferase inhibitor) and panobinostat (a histone deacetylase inhibitor), with chemotherapy with temozolomide (an alkylating agent). The purpose of such combination is to evaluate the use of epigenetic priming to overcome resistance of melanoma to chemotherapy.

**Methods:**

A Phase I clinical trial enrolling patients aged 18 years or older, with recurrent or unresectable stage III or IV melanoma of any site. This trial was conducted with full Institutional Review Board approval and was registered with the National Institutes of Health under the clinicaltrials.gov identifier NCT00925132. Patients were treated with subcutaneous decitabine 0.1 or 0.2 mg/kg three times weekly for 2 weeks (starting on day 1), in combination with oral panobinostat 10, 20, or 30 mg every 96 h (starting on day 8), and oral temozolomide 150 mg/m^2^/day on days 9 through 13. In cycle 2, temozolomide was increased to 200 mg/m^2^/day if neutropenia or thrombocytopenia had not occurred. Each cycle lasted 6 weeks, and patients could receive up to six cycles. Patients who did not demonstrate disease progression were eligible to enter a maintenance protocol with combination of weekly panobinostat and thrice-weekly decitabine until tumor progression, unacceptable toxicity, or withdrawal of consent.

**Results:**

Twenty patients were initially enrolled, with 17 receiving treatment. The median age was 56 years. Eleven (65 %) were male, and 6 (35 %) were female. Eleven (64.7 %) had cutaneous melanoma, 4 (23.5 %) had ocular melanoma, and 2 (11.8 %) had mucosal melanoma. All patients received at least one treatment cycle and were evaluable for toxicity. Patients received a median of two 6-week treatment cycles (range 1–6). None of the patients experienced DLT. MTD was not reached. Adverse events attributed to treatment included grade 3 lymphopenia (24 %), anemia (12 %), neutropenia (12 %), and fatigue (12 %), as well as grade 2 leukopenia (30 %), neutropenia (23 %), nausea (23 %), and lymphopenia (18 %). The most common reason for study discontinuation was disease progression.

**Conclusions:**

This triple agent of dual epigenetic therapy in combination with traditional chemotherapy was generally well tolerated by the cohort and appeared safe to be continued in a Phase II trial. No DLTs were observed, and MTD was not reached.

## Introduction

Until the recent advances in immune and targeted therapeutic approaches, progress in the treatment of metastatic melanoma remained dormant for nearly two decades. The approval of the immune stimulant ipilimumab and the subsequent development of novel targeted agents against BRAF, MEK, and PD-1 have fundamentally changed the landscape of melanoma treatment. Despite the excitement generated by these novel agents, much remains to be understood and significant hurdles remain to be conquered. When individual oncogenic pathways are blocked pharmacologically, melanoma cells find ways to adapt and selectively activate alternative pathways that allow them to “escape” the effects of targeted agents. To prevent this, various trials are evaluating the combined use of drugs targeting multiple pathways simultaneously. While targeting multiple downstream effectors of these pathways might be beneficial, we believe that depriving the cells of the ability to adapt and selectively activate such pathways by targeting upstream epigenetic mechanisms might be a more effective approach.

Epigenetic manipulation is a novel approach to cancer therapy that has proven successful in the treatment of hematologic malignancies, but remains to be further explored in solid tumors. Epigenetic alterations contribute to melanomagenesis by down-regulating tumor suppressor genes, apoptotic mediators, and DNA repair enzymes [1]. They also appear to be an important driving force in resistance mechanisms to multiple therapies. There is evidence that epigenetic silencing may contribute to resistance to chemotherapeutics and that drugs targeting epigenetic mechanisms may enhance chemosensitivity [2, 3]. Epigenetic drugs also appear to enhance the endogenous anti-tumor immune response via several mechanisms including, but not limited to, increased expression of cancer-testis antigens [4–14]. Furthermore, epigenetic drugs have shown the ability of reconstituting the functionality of apoptotic processes that, when deregulated, appear to play a crucial role in the resistance to chemotherapeutics [15], immune responses [11, 16], and targeted agents such as BRAF and MEK inhibitors [17, 18]. These, along with many other potential mechanisms, support the notion that epigenetic modifications represent a global mechanism for treatment resistance in melanoma.

In this Phase I trial, we explore the safety and tolerability of combining two epigenetic drugs: decitabine [a DNA methyltransferase (DNMT) inhibitor] and panobinostat [a histone deacetylase (HDAC) inhibitor], with traditional chemotherapy with temozolomide (an alkylating agent), setting the stage of epigenetic interruption of melanoma cell resistance. This trial started enrolling patients when temozolomide was a standard treatment for metastatic melanoma, prior to the approval of ipilimumab and subsequent targeted therapies. The primary objective of this trial was to evaluate the safety and tolerability of this triple agent regimen at previously defined doses. Since the use of decitabine in this trial was aimed at achieving epigenetic modification and not cytotoxicity, decitabine was administered at low doses known to cause hypomethylation. Panobinostat was dose-escalated as shown in Table 1. Temozolomide was administered at standard doses. While our model tested epigenetic drugs in combination with chemotherapy, we believe that a similar approach could be used with the newer immune and targeted therapies.

**Table 1:** Doses of decitabine and panobinostat^a^

Cohort	Decitabine (subcutaneously, three times weekly for 2 weeks) (mg/kg)	Panobinostat (orally, every 96 h) (mg)	No. of patients
1	0.1	10	5
2	0.1	20	4
3	0.2	20	4
4	0.2	30	4

^a^All cohorts received oral temozolomide at a dose of 150 mg/m^2^/day on days 9 through 13 on cycle 1

## Materials and methods

### Patients and eligibility criteria

Eligible participants included male or female patients that were 18 years of age or older, with recurrent or unresectable stage III or IV melanoma of any site. Since we sought to evaluate enhancement of chemosensitivity by epigenetic drugs, this trial enrolled patients with inherently aggressive and resistant disease, including noncutaneous melanoma like ocular and mucosal; patients with brain metastases, after the brain disease was adequately addressed either by whole brain radiation, radiosurgery, or resection; and patients that had progressed during or after their most recent treatment. Eligibility criteria also included adequate liver, renal, cardiac and bone marrow function; normal electrolytes; normal thyroid function (or on adequate replacement doses); normal LVEF by MUGA or echocardiogram; measurable disease per RECIST 1.0 criteria; and Eastern Cooperative Oncology Group (ECOG) performance status of 0–2. Previously treated or treatment-naïve patients were both eligible, except those who had previously received valproic acid, HSP90 inhibitors, hypomethylating agents, or HDAC inhibitors. Female patients of childbearing potential were required to not be pregnant, breast-feeding, and to use double contraception during and 3 months after study completion.

Exclusion criteria included: uncontrolled hypertension; history of ventricular fibrillation, torsades de pointes, or sustained ventricular tachycardia; heart rate <50 beats/min; congestive heart failure NYHA class III or IV; acute coronary syndrome within 6 months of study enrollment; ECG abnormalities of QTc prolongation (>450 ms), right bundle branch block or left anterior hemiblock; known HIV or hepatitis C positivity; unresolved diarrhea or significant gastrointestinal impairment potentially interfering with panobinostat or temozolomide absorption; and concomitant use of CYP3A4 inhibitors or drugs known to increase risk of torsades de pointes.

This trial was conducted with full Institutional Review Board approval. All participants provided written consent before participating. This study was registered with the National Institutes of Health under the clinicaltrials.gov identifier NCT00925132. Novartis provided panobinostat and financial support for this trial.

### Study treatment and dose escalation

**Fig. 1:**
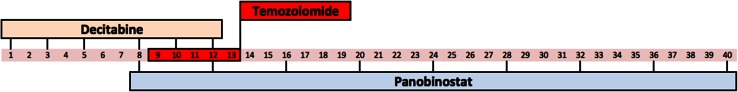
Treatment schema. Cycle duration: 42 days. Decitabine: days 1, 3, 5, 8, 10, 12. Panobinostat: days 8, 12, 16, 20, 24, 28, 32, 36, 40. Temozolomide: days 9–13

Patients were treated with subcutaneous decitabine at a dose of 0.1 or 0.2 mg/kg three times weekly for 2 weeks (starting on day 1), in combination with oral panobinostat at a dose of 10, 20, or 30 mg every 96 h (starting on day 8), and oral temozolomide at a dose of 150 mg/m^2^/day on days 9 through 13 (Fig. 1; Table 1). In cycle 2, temozolomide dose was increased to 200 mg/m^2^/day if neutropenia or thrombocytopenia had not occurred. Prophylactic trimethoprim–sulfamethoxazole was not used. Each treatment cycle lasted 6 weeks, and patients could receive up to six cycles of combination treatment. Patients who did not demonstrate disease progression were eligible to enter a maintenance protocol with combination of weekly panobinostat and thrice-weekly decitabine until tumor progression, unacceptable toxicity, or withdrawal of consent. Maximum tolerated dose (MTD) was defined as the highest dose cohort where ≤1/6 patients experienced a dose-limiting toxicity (DLT). If a DLT was observed in the first three patients, the cohort was expanded to six patients, and all six patients needed to complete the first cycle of therapy without an additional DLT before dose escalation could proceed. Intrapatient dose escalation was not allowed. DLT was defined as grade 4 hematologic toxicity, grade ≥3 nonhematologic toxicity, or grade 2 nonhematologic or grade 3 hematologic toxicity requiring a dose reduction or treatment interruption for more than 7 days during the first cycle. Grade 3 or 4 nausea, vomiting, or diarrhea were only considered DLTs if they occurred despite optimal medical management. Grade 3 electrolyte, uric acid, or phosphorus abnormalities were not considered DLTs if they were correctable within 1 week.

In cycle 2, dose was increased to 200 mg/m^2^/day if there was no neutropenia or thrombocytopenia.

### Safety and response assessments

Patients were assessed for safety every 2 weeks during the first two cycles and then once every cycle. CBC with differential and serum chemistries were obtained once a week during the first two cycles and every 2 weeks thereafter. ECGs were performed prior to and following the first dose of panobinostat during cycle 1, and then on day 8 of every subsequent cycle. Toxicity was graded according to the National Cancer Institute (NCI) Common Terminology Criteria for Adverse Events (CTCAE), version 3.0.

Tumor response was assessed using whole body FDG PET–CT or CT scan after two cycles of treatment. Response was determined based on the response evaluation criteria in solid tumors (RECIST).

## Results

### Patient characteristics

Twenty patients were enrolled in the Phase I portion of this study. One patient had rapid progression of disease and was not treated with the protocol. One patient did not meet eligibility criteria due to untreated brain metastases. One patient withdrew consent. A total of seventeen patients received treatment. Characteristics are listed in Table 2. The median age was 56 years. Eleven (65 %) were male, and 6 (35 %) were female. Eleven (64.7 %) had cutaneous melanoma, 4 (23.5 %) had ocular melanoma, and 2 (11.8 %) had mucosal melanoma.

**Table 2:** Patient characteristics (*n* = 17)

Characteristics	Value (range)
Male:female	11:6
Median age	56 (32–77)
Melanoma location	
Cutaneous	11
Ocular	4
Mucosal	2
Median no. of prior systemic treatments	1 (0–3)
ECOG	0–1
Median no. of cycles administered	2 (1–6)

### Exposure to treatment and clinical toxicities

All seventeen patients received at least one treatment cycle and were evaluable for toxicity. Patients received a median of two 6-week treatment cycles (range 1–6). The dose escalation schema and number of patients enrolled in each cohort are described in Table 1. None of the patients experienced DLT. One patient in cohort 3 had grade 4 neutropenia that resolved within 3 days and did not meet criteria to be categorized as DLT. MTD was not achieved. Adverse events in each cohort are summarized in Table 3. These included grade 3 lymphopenia (24 %), anemia (12 %), neutropenia (12 %), and fatigue (12 %), as well as grade 2 leukopenia (30 %), neutropenia (23 %), nausea (23 %), and lymphopenia (18 %). The most common reason for study discontinuation was disease progression.

**Table 3:** Summary of adverse events

Cohort	Grade 3 (no. of subjects)^a^	Subject^b^	Cycle and day	Grade 4 (no. of subjects)	Subject^b^	Cycle and day	DLT
1	Lymphopenia (1)	#03	C1, D12	None			None
	Anemia (1)	#02	C1, D12				
	Fatigue (1)	#02	C2, D3				
	Nausea (1)	#02	C2, D3				
2	None			None			None
3	Lymphopenia (3)	#12	C1, D40	Neutropenia (1)	#13	C1, D21	None
	Anemia (1)	#13	C1, D40				
	Neutropenia (1)	#14	C1, D15				
	Fatigue (1)	#14	C1, D15				
	Fever (1)	#14	C1, D20				
	Hypokalemia (1)	#15	C4, D18				
	Back pain (1)	#15	C2, D3				
		#15	C3, D5				
		#12	C1, D33				
4	Thrombocytopenia (1)	#20	C1, D25	None			None

^a^No. of subjects: number of subjects that developed the adverse event listed

^b^Subject that developed each adverse event is listed to note that some of the adverse events occurred in the same subject in a given cohort

The majority of the adverse events occurred in cohort 3. Of the 10 adverse events observed, only 8 were deemed to be treatment related (hypokalemia and back pain were not treatment related). Subjects’ characteristics and underlying disease might have contributed to the relatively high occurrence of adverse events in Cohort 3. Two out of the four subjects in cohort 3 were withdrawn from the study due to rapid disease progression and died shortly after.

### Treatment efficacy

Of the 17 patients treated, 9 were considered nonevaluable for efficacy given that they did not complete two cycles of therapy due to early disease progression. Among eight patients evaluable for radiographic response, 6 (75 %) had either stable disease (5, 62.5 %) or complete response (1, 12.5 %). The patient with the complete response had mucosal melanoma and had the best response after two cycles of therapy. Response lasted for 8 months. Of the five patients with stable disease, two were from Cohort 1, two from Cohort 3, and one from Cohort 4. The two remaining patients (25 %) had progressive disease after two cycles of therapy (Table 4).

**Table 4:** Response assessment (by RECIST criteria)

Cohort	Melanoma origin	Type of response
1	Cutaneous	Stable disease
1	Cutaneous	Stable disease
1	Mucosal	Complete response
2	Ocular	Progression
3	Ocular	Stable disease
3	Cutaneous	Stable disease
4	Mucosal	Stable disease
4	Ocular	Progression

## Discussion

The field of epigenetics might offer a novel approach to the treatment of melanoma that could potentially add to the recent progress in immune and targeted therapies. Though significantly explored in hematologic malignancies, epigenetic therapeutic approaches have lagged behind in solid tumors. It has been well established that epigenetic processes, specifically DNA promoter methylation and histone acetylation/deacetylation, are key cellular events during tumorigenesis [19]. Moreover, melanoma cells appear to use the epigenetic apparatus to “adapt” and acquire resistance to external offenders such as chemotherapeutics [15], the immune system [11, 16], and even the newer targeted agents [17, 18]. In this Phase I trial, we explored the safety and tolerability of traditional chemotherapy combined with dual epigenetic therapy with sequential DNMT and HDAC inhibition. We used temozolomide, a known standard agent for metastatic melanoma prior to 2011, combined with decitabine and panobinostat.

During DNA methylation, a methyl group is added to cytosine in CpG islands located predominantly in promoter regions, resulting in the silencing of genes regulated by the affected promoter. Abnormal genetic silencing by DNA methylation appears to modulate cancer biology and development of drug resistance [20]. Decitabine is a powerful DNMT inhibitor that has shown the ability to impair the methylation process in numerous cancer cell lines (including melanoma), allowing the re-expression of genes that malignant cells are trying to turn off [5]. There is evidence that the doses required to achieve hypomethylation are much lower than the usual cytotoxic doses [21–23]. In addition, given that active cell cycling is required to achieve methylation reversal, prolonged courses achieve more hypomethylation than shorter courses [24]. In myeloid neoplasms, extended administration of low doses of hypomethylating agents may result in increased or sustained response rates [25]. When the goal is to achieve and maintain methylation reversal, keeping decitabine toxicity to a minimum might be key, as this allows for repeated doses and longer courses. This approach might be more effective than trying to push decitabine doses until DLT or MTD are reached [20]. Most trials using decitabine in solid tumors have used high, toxic doses, with short administration periods [26–29]. These factors, at least partly, may account for the disappointing responses to decitabine in solid tumors, as compared to responses in hematopoietic malignancies. For these reasons, in this trial, we used low doses of decitabine in an extended dosing regimen. DLT or MTD were not reached, allowing for repeated dose administration.

In addition to promoter methylation patterns, gene expression is also highly influenced by DNA–histone interactions that regulate the ability of the transcription apparatus to access the DNA. The opposing activities of histone acetyltransferase and HDAC maintain histone acetylation patterns that lead to cell-specific gene expression profiles. Aberrant HDAC recruitment appears to play a critical role in gene expression changes seen in malignant transformed cells that allow them to block apoptotic mechanisms. HDAC inhibitors appear to reestablish apoptosis in melanoma cells [30], induce cell differentiation, and inhibit tumor growth in animal models by down-regulating positive cell cycle regulators such as cyclin D1, c-Myc, C-RAF, and AKT [31–37], while inducing the expression of a number of anti-proliferative genes [38–40]. Melanoma cells exposed to HDAC inhibitors also exhibit decreased levels of activated MEK1/2 and ERK1/2 [41], key melanomagenic kinases blocked by novel targeted agents. HDAC inhibitors may also interfere with the appropriate folding of HSP90-client proteins (including AKT and RAF) that are critical to cancer cell growth [36, 37]. HDAC inhibitors, however, are unable to reactivate the expression of genes that have been previously silenced by methylation of their promoters. This provides a rationale for the sequential use of DNMT inhibitors followed by HDAC inhibitors to provide “epigenetic synergy,” which has shown to enhance gene re-expression and drug sensitivity [42]. In this trial, we administered Panobinostat, a novel and potent HDAC inhibitor, a week after decitabine initiation. In animal studies, panobinostat has been shown to have affinity to melanin, judged by measurable drug-related radioactivity in the uveal and pigmented skin at 96 h port-dose administration (data from Novartis Investigator’s Brochure). In our study, panobinostat was administered every 96 h.

This triple agent regimen of decitabine, panobinostat, and temozolomide was generally well tolerated by the cohort and appeared safe to be continued in a Phase II trial. No DLTs were observed, and MTD was not reached. As discussed before, when the goal is to achieve epigenetic modulation, administration of higher doses of DNMT inhibitors might hinder the ability of patients to tolerate the frequent dosing intervals required to maintain hypomethylation throughout the treatment cycle. Maintaining hypomethylation also appears to be more important than deepening the nadir of methylation in each cycle. Given that the doses used in all cohorts proved safe, cohort 3 dose level was the recommended dose for the Phase II portion of this trial, as this is the subcutaneous dose of decitabine that has been shown to achieve successful hypomethylation [43]. This is supported by the observation of a gradual increase of hemoglobin F concentration 2 weeks after the initiation of therapy (preliminary data from Phase II, not shown) that appears to persist through the course of treatment. Patient responses in this cohort as shown in Table 4 are intriguing, especially given the inclusion of patients with ocular and mucosal melanoma, two highly chemoresistant variants of melanoma. The complete response observed in one subject with mucosal melanoma is very intriguing. This subject achieved this best response (complete response) after two cycles. Mucosal melanomas tend to have c-kit mutations and are generally negative for B-RAF. They metastasize quite frequently, behaving differently from cutaneous melanoma. The effect of epigenetic therapy in this patient population might provide clues to the disease biology and warrants more investigation. However, the rarity of these tumors will likely make studying this subpopulation more difficult. We hypothesize that DNMT inhibition followed by HDAC inhibition target key epigenetic events that melanoma cells use to selectively turn on or off specific pathways that confer resistance to chemotherapy and apoptosis. This study started enrolling patients prior to the approval of ipilimumab and the new targeted agents that are revolutionizing the treatment of melanoma. We believe that epigenetic alterations might represent a global resistance mechanism in melanoma and other cancers. Clinical trials using a similar approach, but this time combining epigenetic agents with immune therapies and novel targeted agents such as BRAF, MEK, or PD-1 inhibitors, are warranted. This approach of crippling “upstream” epigenetic mechanisms that allow melanoma cells to adapt and acquire resistance to novel agents could prove to be an alternative to blocking multiple “downstream” effectors using multiple targeted agents.
